# The Stimulatory Effect of Purine-Type Cytokinins on Proliferation and Polyphenolic Compound Accumulation in Shoot Culture of *Salvia viridis*

**DOI:** 10.3390/biom10020178

**Published:** 2020-01-24

**Authors:** Izabela Grzegorczyk-Karolak, Katarzyna Hnatuszko-Konka, Mariola Zarzycka, Łukasz Kuźma

**Affiliations:** 1 Department of Biology and Pharmaceutical Botany, Medical University of Lodz, Muszynskiego 1, 90-151 Lodz, Poland; mariola.zarzycka@stud.umed.lodz.pl (M.Z.);; 2 Department of Molecular Biotechnology and Genetics, Faculty of Biology and Environmental Protection, University of Lodz, Banacha 12/16, 90-237 Lodz, Poland; katarzyna.hnatuszko@biol.uni.lodz.pl

**Keywords:** benzylaminopurine riboside, N-benzylotetrahydropyranyl adenine, meta-topoline, rosmarinic acid, verbascoside

## Abstract

The present study demonstrates hormonal control of *Salvia viridis* growth and development using four different purine-type cytokinins at different concentrations. The addition of cytokinins significantly increased biomass of cultures, proliferation rate, and, interestingly, secondary metabolite production. The best response in terms of multiplication ratio was recorded on Murashige and Skoog medium supplemented with 0.5 mg/L BPA (N-benzylotetrahydropyranyl adenine), while the greatest biomass accumulation was achieved when supplemented with 1 mg/L *m*-T (meta-topoline). Quantitative UPLC-DAD analysis of the hydromethanolic extract from *S. viridis* culture revealed the presence of 12 polyphenols: seven phenolic acids and five phenylethanoids. The highest total content of polyphenolic compounds was found in shoots cultivated on medium with 2 mg/L BPA (18.66 mg/g DW): almost twice that of control shoots. The medium was also the most optimal for the biosynthesis of rosmarinic acid, the predominant phenolic acid. However, the greater phenylethanoid accumulation was stimulated by 1 mg/L *m*-T: the metabolite content was above three times higher than that found in shoots grown on the control medium (8.03 mg/g DW vs. 2.37 mg/g DW). Hence, it was demonstrated that phytohormones are capable of influencing not only vital physiological processes, but therapeutic potential of plants as well. Therefore, the cytokinin-based sage cultures may be also considered as the alternative sources of bioactive compounds.

## 1. Introduction

*Salvia viridis* L. (synonym *S. horminum* L.) is currently cultivated in Europe mainly as an ornamental plant. However, it once played a significant role in traditional medicine. Infusions of sage leaves were mainly used in inflammation of the oral cavity, gums, and throat [1,2]. The plant extracts were also used to rinse the eyes to treat inflammation, and viral and bacterial infections [3]. Various phenolic acids, flavonoids, phenylethanoids, triterpenes, and phytosterols have been isolated from the aerial parts of *S. viridis* [1,2,4]. The in vitro grown shoots of *S. viridis* have been found to be valuable sources of bioactive phenolic compounds [5], with the hydromethanolic extract of obtained shoot culture being especially rich in rosmarinic acid and verbascoside: metabolites known for numerous health-promoting activities. Rosmarinic acid (RA), a phenolic compound naturally-occurring in a number of Lamiaceae family plant, is a promising therapeutic agent against a wide variety of civilization diseases. It exhibits antioxidant, anti-inflammatory, anticancer, antimicrobial, antidiabetic, cardiprotective, neuroprotective, and even antidepressant activities [6]. Similarly, verbascoside possesses numerous pharmacologically beneficial activities, including antioxidant, anti-inflammatory, neuroprotective, antibacterial, antifungal, and antineoplastic properties [7].

Biotechnological methods could be used to obtain a significant amount of valuable raw material in a short time. They make it possible to carry out cultivation independent of climatic conditions and the ability of annual plants, such as *S. viridis*, to produce seeds and germinate. Therefore, there is great interest in developing an effective method of *S. horminum* proliferation on artificial media in in vitro conditions. The choice of growth regulators plays a key role in such media by affecting the physiological processes taking place in the plant. By using plant hormones in culture, it is possible to inhibit or induce growth, shoot formation or metabolite biosynthesis pathways.

The aim of the work was to optimize the process of propagation of *Salvia viridis* shoots by examining changes in growth rate and phytochemical accumulation in *S. viridis* shoot culture in response to treatment with different cytokinin types and concentration. The culture was carried out on media supplemented with four selected cytokinins, *viz.* benzyloaminopurine (BAP), benzylaminopurine riboside (rBAP), N-benzylotetrahydropyranyl adenine (BPA) and meta-topolin (*m*-T), at three concentrations: 0.5, 1.0 and 2.0 mg/L. Phytochemical quantification was carried out by high performance liquid chromatography.

Several species of sage have been successfully propagated in vitro in the presence of various natural and synthetic cytokinins, derivatives of purine and urea. The growth regulators chosen for the present study are based on a purine skeleton. This choice was not accidental, as it has been found that cytokinins possessing a heterocyclic system in their structure gave better results in the multiplication of *Salvia* species. Media containing 0.5 mg/L benzylaminopurine, were found to yield a multiplication atio for *Salvia guaranitica* 2.5 times that of a culture treated with the same TDZ (thidiazuron) content [8]. BAP was also more effective than thidiazuron in the proliferation of *Salvia brachyodon* [9], *Salvia nemorosa* [10], and *Salvia fruticosa* [11]. Based on these studies, and those performed on *Salvia africana-lutea* [12] or *Salvia virgata* [13], MS medium supplemented with auxin and 0.5 mg/L BAP was used for the establishment of *S. viridis* culture, and its initial proliferation [5]. However, this protocol for the development of sage shoots revealed that this combination of growth regulators did not give satisfactory multiplication results (only 1.3 shoot per explant). On the other hand, this culture has been found to display great potential for bioactive compound production. Hence, the investigation on the composition of the medium could result in the optimization of both, the proliferation rate and therapeutic value. Therefore, subsequent studies examined the use of other concentrations, as well as other cytokinins from the purine group in the growth medium.

## 2. Material and Methods

### 2.1. Plant Material

The shoot culture was established using *Salvia viridis* seeds obtained from the Garden of Medicinal Plants of Wroclaw, Poland. The procedure of seed sterilization, their germination in in vitro condition and development of primary culture were described earlier [5]. Voucher specimens (no. IG/SVS1-4/2017) of the plants were deposited in the Department of Biology and Pharmaceutical Botany, Medical University of Lodz. The explants used in the experiment were obtained from shoot culture grown on agar solidified (0.7%) MS (Murashige and Skoog) medium [14] with 0.1 mg/L IAA (indole-3-acetic acid) and 0.5 mg/L BAP (benzyloaminopurine). 

### 2.2. Shoot Proliferation and Biomass Accumulation 

The single shoot tips of 5 week-old aseptically in vitro grown shoots were cultivated on MS medium supplemented with 0.1 mg/L IAA and various purine-type cytokinins: BAP, *m*-T, BPA or rBAP at different concentrations: 0.5, 1, or 2 mg/L. All the growth regulators were obtained from Duchefa Biochemie (Haarlem, The Netherlands). Explants grown on MS medium with 0.1 mg/L IAA without cytokinins (C) were used as controls. The inoculum fresh weight was 0.024 g and dry weight 0.0031 g. The cultures were cultivated in glass tubes containing 25 mL of medium. The shoots were grown under a 16-h photoperiod provided by cool white fluorescent lamps (40 μmol m^−2^ s^-1^), at 26 ± 2 °C. The experiments were repeated three times, and each series consisted of about 10 explants.

After five weeks, the multiplication rate, i.e., the mean number of new buds (<0.5 cm long) and/or shoots (≥0.5 cm long) on an explant, the mean length (cm) of the main shoot and obtained shoots, shoot morphology, their fresh (FW) and dry weight (DW) [g/culture] were recorded.

### 2.3. Determination of Polyphenolic Compounds

Briefly, 100 mg of lyophilized plant material obtained from cultivation on media treated with different types and concentrations of cytokinins were extracted according to Grzegorczyk-Karolak et al. [5]. Quantitative chromatographic analysis was performed with an Agilent Technologies 1290 Infinity UPLC as described previously [15]. Separations were performed on a Zorbax SB C18 column (Agilent, Lexington, MA, USA) operating at 25 °C at a flow rate of 0.2 mL/min. The compounds were identified by comparing their retention time, MS spectra and UV spectra with those of the standard and/or published data as reported earlier [4,5]. The total content of compounds was calculated by summing up the contents of individual quantified compounds. The compound levels were expressed in mg per g DW.

### 2.4. Statistical Analysis

Means and standard errors (SE) were calculated using MS-Excel 2010. Analysis of variance was performed using one-way ANOVA, followed by the post hoc Tukey’s test. The analysis was performed with STATISTICA 10.0 software (Statsoft Inc., Krakow, Poland). The significance level was assumed to be 5%.

## 3. Results

### 3.1. Effect of Purine-Type Cytokinins on Shoot Propagation and Biomass Accumulation

**Figure 1:**
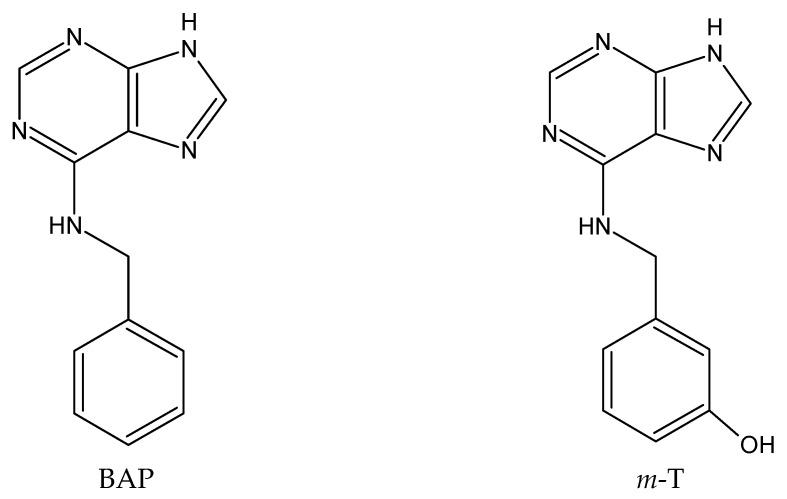

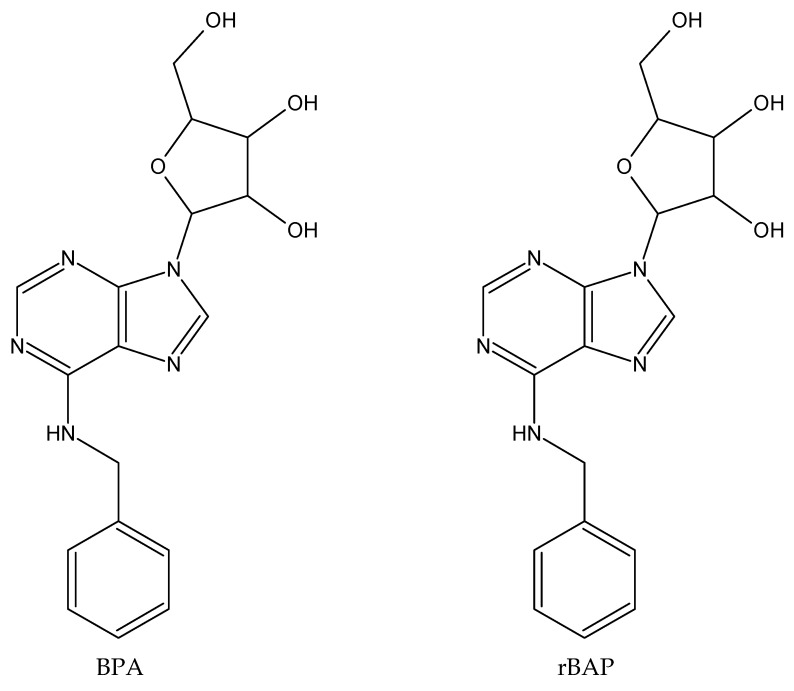
Chemical structures of purine-type cytokinin used in the study.

The shoots of *S. viridis* were grown on solid MS medium supplemented with four different cytokinins (BAP, *m*-T, BPA, rBAP) (Figure 1) at different concentrations (0.5, 1 or 2 mg/L). Analyzing all growth parameters, it was found that the type of cytokinin in the medium and its concentration had a significant effect on shoot propagation.

**Figure 2:**
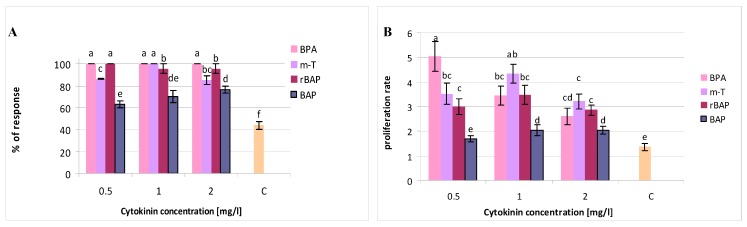

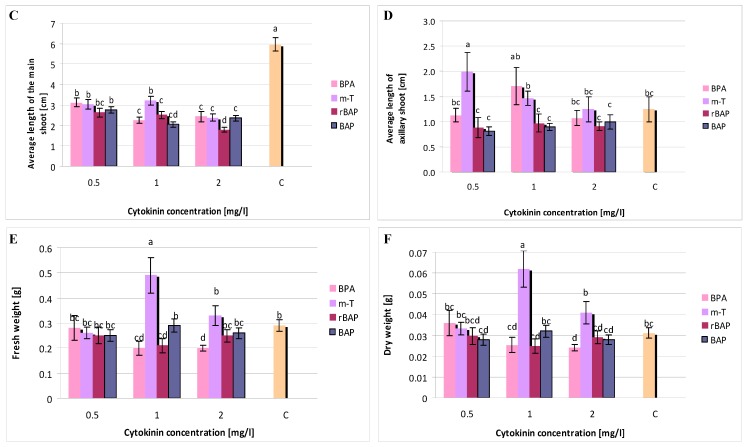
Effect of cytokinin type and concentration on: (**A**) proliferative response (%), (**B**) proliferation rate, (**C**) length of the main shoot, (**D**) length of the axillary shoots, (**E**) fresh weight, and (**F**) dry weight of culture of *S. viridis.* C- control, shoots cultivated on MS medium only with auxin (0.1 mg/L IAA). The values represent the mean ± standard error of three independent experiments. Means marked with the same letter were not significantly different according to the one-way ANOVA test, followed by the post hoc Tukey’s test for multiple comparison (*p* ≤ 0.05).

The multiplication response of cultures grown on media with cytokinins, expressed as new bud and/or shoot formation, ranged from 63% to 100% (Figure 2A). The lowest response was obtained from culture grown on media with BAP. However, the values were also significantly higher than on the control medium (44%). Initially, a medium without plant growth regulators was used as a control. However, in this case, the shoots were characterized by not only a low response (13.7%) and proliferation ratio (1.3), but their shoot tips began to darken and a large number of plants withered and died before the fifth week of cultivation. Supplementing the medium with auxin did not improve the proliferation rate, but increased the viability of the shoots and stimulated their growth. The greatest length was achieved by shoots grown on a medium with 0.1 mg/L IAA but without cytokinins (6 cm); these were two or three times longer than those cultured with the growth regulators (Figure 2C). There is a well-known auxin tendency to promote shoot elongation due to stimulation of apical dominance in shoot culture. The effect of cytokinins is the opposite, which is why their present in the medium, especially at a high concentration, can inhibit shoot elongation [16].

**Figure 3:**
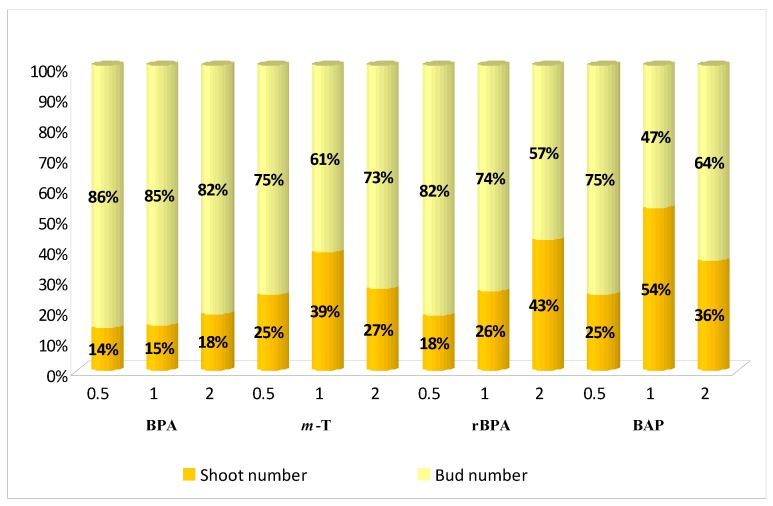
Ratio of shoots to buds of *S. viridis* formed on MS medium with 0.1 mg/L IAA and different cytokines at different concentrations.

Furthermore, all shoots grown on treated media, for all types and concentrations of cytokinins, excluding BAP at a concentration of 0.5 mg/L, demonstrated a higher multiplication rate than control (Figure 2B). The highest proliferation level was found in the case of shoots cultivated in the presence of 0.5 mg/L BPA (5). An increase in BPA concentration caused a decrease in the multiplication factor; it reduced to half the value (2.9) at the highest concentration (2 mg/L). Although the mean number of buds/shoots obtained per explant varied significantly depending on the concentration of BPA, the ratio of the received shoots to buds was approximately 15:85 for all concentrations (Figure 3). Hence, within five weeks, mainly small buds were obtained on explants, and these required an additional passage to lengthen them before they could be used in the next stages of micropropagation. Extending the cultivation time did not enhance bud development. insignificant bud growth was observed during the following (sixth) week, accompanied by the withering and dying of apical parts or even whole shoots. This effect was sometimes reported during the fifth week for shoots grown on control medium and on media supplemented with BAP, especially at lower concentrations.

MS medium with 1 mg/L *m*-T proved to be the most efficient for *S. viridis* shoot growth. Although the cultures demonstrated a slightly lower multiplication ratio (4.4; without statistically significant difference) than for culture grown on medium with 0.5 mg/L BPA (Figure 2B), they nevertheless demonstrated a much higher participation of shoots among the obtained structures (40:60) (Figure 3). They also obtained the highest culture FW (0.28 g) and DW (0.036 g/culture) (Figure 2E,F), this being a 20-fold increase in culture biomass over five weeks; the medium with 0.5 mg/L BPA demonstrated an 11-fold increase. Sage shoots grown on medium with 1 mg/L *m*-T achieved great length (3.2 cm) (Figure 2C) and the length of the axillary shoots was also above average (1.5 cm) (Figure 2D). A decrease to 0.5 mg/L or an increase to 2.0 mg/L of *meta*-topoline concentration in the culture medium resulted in a decrease in the multiplication ratio to approx. 3.5 (Figure 2B), a decrease in the response percentage from 100% to 85% (Figure 2A), and a reduction in the length of the main shoot and the ratio of the number of obtained shoots to buds; however, this parameter was still more favorable for all cultures grown in media with *m*-T than in those treated with the other cytokinins used in the experiment (Figure 3). Besides, shoots growing on media with the addition of *m*-T, irrespective of its concentration, formed good quality buds and shoots with normal morphology. The change in *m*-T concentration in the medium also resulted in a decrease in culture FW and DW.

Benzylaminopurine riboside, despite giving a high percentage of response in the form of new buds/shoots on explants (95%–100%) (Figure 2A), offered less favorable proliferation rate (Figure 2B), shoot length (Figure 2C) and culture biomass (Figure 2E,F).

Interestingly, the growth regulator, most commonly-used in the propagation of plants, benzylaminopurine, also widely used in the proliferation of sage species, proved to be the least beneficial in the case of the *S. viridis* culture. Of all the BAP concentrations used, the higher ones (1 and 2 mg/L) proved to be the most beneficial for shoot proliferation. However, the multiplication rate (2) obtained under these conditions was not particularly high and only slightly differed from those obtained for shoots growing on the control media (Figure 2B).

### 3.2. Effect of Purine-Type Cytokinins on Metabolite Production

**Figure 4:**
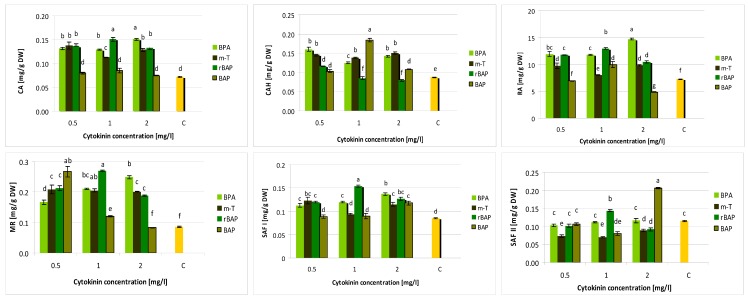
Effect of cytokinin type and concentration on polyphenolic acid content (mg/g DW) in shoot culture of *S. viridis.* CA—caffeic acid, CAH—caffeic acid hexoside, RA—rosmarinic acid, MR—methyl rosmarinate, SAF I—salvianolic acid F (I), SAF II—salvianolic acid F (II), C—control, shoots cultivated on MS medium only with auxin (0.1 mg/L IAA). The values represent the mean ± standard error of three independent experiments. Means marked with the same letter were not significantly different according to the one-way ANOVA test, followed by the post hoc Tukey’s test for multiple comparison (*p* ≤ 0.05).

**Figure 5:**
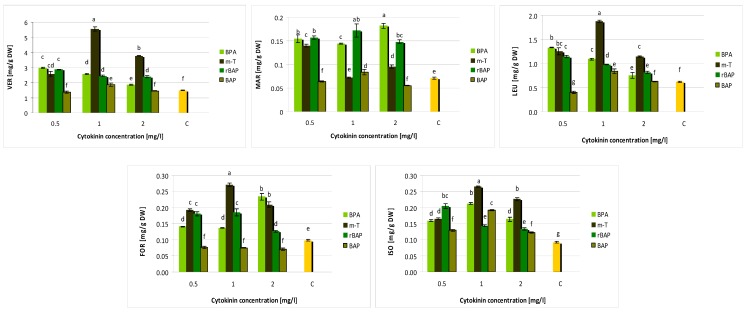
Effect of cytokinin type and concentration on phenylethanoid content (mg/g DW) in shoot culture of *S. viridis.* VER—verbascoside, MAR—martynoside, LEU—leucosceptoside A, FOR—forsythoside A, ISO—isoverbascoside; C—control, shoots cultivated on MS medium only with auxin (0.1 mg/L IAA). The values represent the mean ± standard error of three independent experiments. Means marked with the same letter were not significantly different according to the one-way ANOVA test, followed by the post hoc Tukey’s test for multiple comparison (*p* ≤ 0.05).

UPLC-PDA-ESI-MS/MS analysis of the hydromethanolic extract from *S. viridis* shoots grown on media with different growth regulator combinations identified twelve compounds. The compound content was evaluated using UPLC method as described by Grzegorczyk-Karolak et al. [5]. Polyphenolic levels proved to be dependent on the type and concentration of cytokinins in the medium (Figure 4 and Figure 5). Two groups of compounds were identified: five phenylethanoids and seven polyphenolic acids; however, salvianolic acid B was only indicated in trace amounts in some extracts. Generally, phenolic acids predominated in the extracts, but the ratio of total phenolic acid content to total phenylethanoid content ranged from 1.1 to 4.8, depending on the type and concentration of cytokinin present in the medium (Table 1).

The predominant metabolite in all analyzed extract was rosmarinic acid. Its content ranged from 5.6 to 14.7 mg/g DW (Figure 4). The maximum amount of the compound was found in shoot cultivated on medium with 0.1 mg/L IAA and 2 mg/L BPA. The highest BPA concentration was also very effective for the production of other compounds from the group of polyphenolic acids, yielding the highest total level of phenolic acids (15.5 mg/g DW) and total polyphenolic compound content (18.7 mg/g DW) in *S. viridis* shoots (Table 1). However, the highest tested BPA concentration was unfavorable for phenylethanoid production, resulting in the highest total polyphenolic acid to phenylethanoid ratio (4.8). Lower BPA concentration were associated with lower RA and total polyphenolic compound content.

Shoots grown on a medium supplemented with rBAP and *m*-T were also characterized by high polyphenolic compound production; however, lower levels of rosmarinic acid (below 10 mg/g DW) and total polyphenolic acids (8.6–10.7 mg/L) were found in the presence of *m*-T. On the other hand, *m*-T intensively stimulated the production of phenylethanoids (Figure 5). At the optimal concentration of *m*-topoline (1 mg/L), the extract contained approximately 5.5 mg verbascoside per g DW and 1.9 mg of leucosceptoside. Approximately 16.7 mg/g DW polyphenolic compounds were obtained in the extract from shoots grown on the medium with 1 mg/L *m*-Top; however, almost half this amount was composed of phenylethanoids (8.03 mg/g DW) (Table 1).

BAP as one did not prove to be beneficial for the production of polyphenolic compounds in *S. viridis* shoot culture. The media with the lowest (0.5 mg/L) and the highest (2 mg/L) BAP content induced lower production of polyphenolic compounds in shoots than the control MS medium with auxin alone. Medium supplemented with 1 mg/L BAP demonstrated significantly higher production of rosmarinic acid and other phenolic compounds (TPC = 13.6 mg/g DW), but even in this case, benzylaminopurine was less effective than other cytokinines regardless of their concentration (Figure 4 and Figure 5).

## 4. Discussion

The presented study demonstrates hormonal influence of using four different purine-type cytokinins at different concentrations on shoots of *Salvia viridis*. The addition of cytokinins significantly increased biomass of cultures, proliferation rate and, what interesting, secondary metabolite production. Hence, the results are discussed at two complementary levels. Firstly, at level of the cognitive analysis that demonstrates the correlation between the chemical structure-dependent metabolism of cytokinins and the biochemical processes in the sage culture. Secondly, the research results underline the possibility of pharmacological application of the rich in bioactive metabolites raw material.

Of all the cytokinins employed for micropropagation procedures, BAP is the most widely-used due to its effectiveness and affordability. However, the use of inappropriate types or concentrations of cytokinin can have negative effects on proliferation, mass accumulation and the quality of shoots in some plant species [17]. BAP has been shown to be more likely to induce hyperhydricity in some species compared to other cytokinins: a morpho-physiology disorder observed in in vitro propagated shoots under the influence of used cytokinins, especially at high concentrations.

Hence, these limitations have spurred the search for new plant growth regulators. These searches are becoming increasingly focused because of the increasing knowledge of the metabolism and interactions of these molecules. When supplemented to the medium, free cytokinins can be converted into their corresponding nucleosides and nucleotides, which are highly active; however, they can be further transformed by N7- and N9-glucosylation into biologically-inactive compounds. One way of prolonging the effect of cytokinin can be to use BAP derivatives which are conjugated at the N9-position, i.e., by a ribose in rBAP or a tetrahydropyranyl group in BPA (Figure 1), and hence, resist degradation by N9-glucosylation: this will slow their metabolism in the plant compared to BAP, resulting in a more effective action [17].

The effect was evident in the present study with *S. viridis* shoots, in which BPA and rBAP were used alongside benzylaminopurine. The compounds have not previously been used to multiply *Salvia* species. Chalupa [18] reported using BPA for the growth of *Tilia platyphyllos* shoot culture. When applied at 0.6 mg/L, this cytokinin demonstrated a more effective proliferation than BAP (multiplication rate 4 vs. 3). Supplementation with 2.0 mg/L BPA was optimal for propagating *Vicia faba* [19]. Of the three BPA concentrations used in the present experiment, the lowest (0.5 mg/L) turned out to be the most beneficial for *S. viridis* multiplication: the proliferation rate was three times higher than when BAP was added at the same concentration (Figure 2B). On the other hand, despite being much more effective in the proliferation of *S. viridis* than benzylaminopurine, rBAP proved to be less effective than *m*-T or BPA. On the contrary, rBAP was previously as effective as BPA or *m*-T in the proliferation of *Spathiphyllum floribundum* shoots [20].

Another alternative to modifying the structure of BAP, and thus its activity, could be by introducing hydroxy moieties in the molecule and obtaining hydroxylated BAP analogues. *M*-T, 6-(3-hydroxylbenzylamino) purine (Figure 1), was first isolated from the leaves of *Populus × canadensis* [21]. The hydroxyl group in the *meta*-topoline molecule allows the formation of *O*-glucoside metabolites [20], which are considered to be stable forms of the cytokinin intended for storage; these derivatives can rapidly convert to active cytokinin when required. Such reversible sequestration of *O*-glucosides allows for cytokinin to be made available at a physiologically-active level over a prolonged period of time, resulting in a better response of culture [22]. It has been shown that the slight structural differences between BAP and *m*-T have significant effects on *S. floribundum* cultures, probably due to their distinct metabolism in plant tissues: BPA and rBAP, like BAP, were mainly converted into the stable derivative N6-benzylaminopurino-9-glucoside located at the basal part of the plant, meanwhile N6-(3-*O*-glucopyranosyl) benzylainipurine-9-riboside was the main derivative of *m*-T [20].

Reports describing the use of *m*-T in plant biotechnology are not numerous, but they are very promising. According to published data, *meta*-topoline stimulates the transformation of buds into shoots, improves the morphology of the obtained structures and increases the survival of obtained shoots and plants. Bairu et al. [23] found that plants of *Aloe polyphylla* treated with *m*-T were superior in quality and quantity compared to those treated with BAP. In this experiment, *m*-T was found to be a good replacement for zeatin, despite the real structural differences between them. Mok et al. [24] found the *Arabidopsis* AHK4 receptor to respond in the same way to trans-zeatin and *m*-T. This similar affinity in receptor recognition, despite the differences in structure, may be the reason why *m*-T and zeatin demonstrated comparable effects in *A. polyphylla* tissue culture.

Shoots of *Pelargonium sidoides* grown in the presence of 0.25 mg/L *m*-T were characterized by a high multiplication ratio (over 11) and the highest biomass increase (178 mg FW and 10.6 mg DW) [25]. Similar observations were described by Aremu et al. [26] and Bairu et al. [23] in relation to several *Musa* ssp. *Meta*-topoline strongly stimulated the proliferation of banana shoots, and even at very high concentration (7.0 mg/L); in addition, it was not toxic for the culture, unlike BAP, which induced an abnormal morphology in the obtained shoots. For two species of the *Prunus* genus, cultures grown on medium with a hydroxylated benzylaminopurine derivative had twice as much fresh weight than those on a medium supplemented with BAP [27]. Although the effect of *m*-T on the propagation of sage species has not been described, it was found to stimulate the proliferation of two other species of Lamiaceae, *Ocimum basilicum* [28] and *Dracocephalum forrestii*, [29] and improve the morphology of the shoot culture. Of three concentrations of *m*-T used in our study, 1 mg/L was found to be most effective. This concentration was also optimal for propagating seven varieties of geranium [30].

While a number of studies have evaluated the efficacy of “new” cytokinins on plant culture proliferation, far fewer have evaluated their effect on the chemical content of proliferated shoots. Polyphenol production is known to have a strong effect on plant growth, as these compounds play an important role in the physiological processes that occur in the plant. At the same times, secondary metabolites are multifunctional bioactive agents. Therefore, the advantages associated with the use of plant growth regulators for the modification of secondary metabolite production in plant culture could be of great interest to researchers.

Another important finding of the presented work is that plants under optimized cytokinin conditions contained significantly increased levels of the pharmacologically active compounds. It is worth noting, that no previous studies have reported the effects of *m*-T, BPA or rBAP on metabolite production in sage shoot culture. However, the effects of BAP and other purine-type cytokinins on bioactive polyphenol accumulation have been described in different plant species. Following supplementation with 0.1 mg/L IAA and 0.45 mg/L BAP, *Salvia officinalis* shoots grown on MS medium produced 16.3 mg RA per g DW, which was similar to that found in the aerial parts of the plant cultivated in natural condition [31]. It has previously been found that BAP stimulated more effective biomass accumulation and RA and SAB production than zeatin, kinetin or TDZ in *Dracocephalum forrestii* shoot culture; however, higher BAP concentrations (8 and 16 µM) were the most optimal for growth, and lower ones (2 µM) were better for production [32]. In contrast, Santos-Gomes et al. [33] report that kinetin yielded greater amounts of RA and CA in *S. officinalis* culture than BAP.

The current findings demonstrate that the type and concentration of cytokinin influenced the levels of the quantified phenolic compounds in the *S. viridis* culture. The phytochemical profile of the in vitro grown shoots was significantly different to that observed in naturally growing plants; they were very rich in flavonoids and phenylethanoids, and contained only small amounts of polyphenolic acids (total phenolic acid content = 1.9 mg/g DW) [4]. Although *S. viridis* shoot cultures are primarily a source of polyphenolic acids, and to a lesser extent also phenylethanoids, the culture undergoes some modification with age, and the phytochemical profile of young, i.e., semi-annual and annual, cultures [5] being different to those of the older (2.5 to three-year-old) cultures described in the present work. For shoots grown on a medium with 0.5 mg/L BAP, on which the shoots were initially cultivated, the level of polyphenols decreased with time [5]: from 15.5 mg/g DW for a six-month culture, through 12.5 mg/g DW for a year-old culture to 9.8 mg/g DW for two-year-old and older cultures. This is associated with a decrease in the production of all compounds from this group. On the other hand, after two years, the phenylethanoid content in the shoots increases. In the presented study, as well as verbascoside and martynoside, three other compounds were identified: leucosceptoside A, forsythoside A, and isoverbascoside. While none of these three were detected in shoot culture younger than one year, they have previously been described in *S. viridis* shoots grown in field conditions [4]. These observations suggest that the changes that occur with age in shoots grown in vitro are similar to those observed in the shoots of the parent plants, particularly regarding the presence of *m*-T in the medium.

Our findings revealed that BAP derivatives have greater stimulatory potential on phenolic content in *S. viridis* shoots than BAP. Although all BAP-derivatives increase polyphenol production, BPA and rBAP displayed a stronger effect on RA and other polyphenolic acid compound pathways, while *m*-T induced higher phenylethanoid production. The greatest RA level was noticed in shoots cultivated on medium containing 2 mg/L BPA, which was 12 times higher than the level observed in aerial parts of plants grown in field conditions [4]. Interestingly, the lowest RA level found in *S. viridis* culture (5.6 mg/g DW) was four-times higher than in shoots of parent plants, highlighting the advantage of the in vitro environment for RA production.

Meanwhile, the amount of verbascoside obtained under optimal conditions in *S. viridis* shoot culture (MS medium with 1 mg/L *m*-T) was 30% lower than in the extract from aerial parts of field-cultivated plants [4]. Of the tested phenylethanoids, only the content of leucosceptoside A was higher in the presented culture than in the hydromethanolic extract of field-grown plant shoots. However, *m*-T clearly stimulated the biosynthesis of verbascoside and other phenylethanoids to a greater degree than other cytokinins: 1 mg/L of *m*-T increased verbascoside to a 3-fold higher level compared to controls. Although no study has described the use of topolins for bioactive compound production in the micropropagated *Salvia* shoots, *m*-T has been found to increase total phenolic, flavonoid and proanthocyanidin content in the aerial parts of regenerated banana plantlets [34]. Amoo et al. [35] also report significantly higher levels of total phenolics, flavonoids and tannins in regenerated shoots of *Aloe arborescens* obtained on medium supplemented with *m*-T than BAP; however, the beneficial effects of *meta*-topoline are not apparent for all secondary metabolites, as the shoots regenerated with BAP produced higher iridoid levels than those with *m*-T. In addition, BAP and *m*-T supplementation were found to demonstrate similar effects on total phenolic and flavonoid accumulation in *Pelargonium sidoides* culture [25]. Contrary to our results, Aremu et al. [36] report that 1 µM *m*-T stimulated less than BAP the production of a number of polyphenolic acids, including protocatechuic acid, 4-hydroxybenzoic acid, vanillic acid, p-coumaric acid, caffeic acid and ferulic acid, in the shoots of *Merwilla plumbea*.

In transformed shoots of *D. forrestii*, cultivation on MS medium with 5 mg/L BPA was found to yield the highest levels of rosmarinic acid, while treatment with 0.2 mg/L *m-*T resulted in the greatest proliferation [29]. Plant growth regulators often intensify or inhibit the biosynthesis of bioactive metabolites in cultivated shoots, and the results do not often correlate with culture growth. BAP is known to stimulate *Scutellaria alpina* shoot growth; it was also more effective at stimulating verbascoside biosynthesis than kinetin and zeatin, but slightly worse than TDZ used at higher concentration [37]. For *Knautia sarajerensis* shoot culture, the highest RA and CA content was observed on medium containing 2 mg/L BAP with these levels being twice those observed for 2 mg/L zeatin; however, the shoots cultivated with zeatin displayed twice the proliferation ratio and shoot growth rate as in presence of BAP [38].

## 5. Conclusions

The experiment attempted to develop an optimal medium for the proliferation of *Salvia viridis* shoots. All phytohormones used in the experiment enabled normal growth and proliferation of sage shoot tips at all used concentrations. The addition of 0.5 mg/L BPA turned out to be the most beneficial in terms of propagation, with a multiplication factor greater than five. However, 1 mg/L *m*-T induced greater increases in culture biomass and shoot to bud ratio. The plant material obtained could be used for further propagation, or as a source of bioactive compounds. Purine-type BAP derivatives have been shown to demonstrate potential in the accumulation of biomass and bioactive polyphenolic compounds in *S. viridis* shoots. Under optimal conditions, compared to the aerial parts of one year-old plants growing under field conditions, *S. viridis* shoots demonstrated 12-times higher RA levels, and eight-times higher total polyphenolic acid levels. Bearing in mind the health-promoting properties of some of these metabolites, *S. viridis* shoot culture appears to offer great potential as a functional food.

## Figures and Tables

**Table 1:** Total polyphenol, phenolic acid and phenylethanoid levels in shoot culture of *S. viridis*.

Cytokinin Type and Content	TPC	TPA	TP	TPA/TP Ratio
C	10.06 ± 0.10	7.69 ± 0.07	2.37 ± 0.03	3.2
BPA 0.5	17.39 ± 0.56	12.62 ± 0.50	4.77 ± 0.06	2.7
BPA 1	16.66 ± 0.17	12.53 ± 0.12	4.13 ± 0.06	3.0
BPA 2	18.66 ± 0.27	15.47 ± 0.17	3.19 ± 0.10	4.8
*m*-T 0.5	14.80 ± 0.81	10.67 ± 0.58	4.12 ± 0.24	2.6
*m*-T 1	16.67 ± 0.25	8.64 ± 0.07	8.03 ± 0.18	1.1
m-T 2	15.94 ± 0.16	10.49 ± 0.11	5.45 ± 0.05	1.9
rBAP 0.5	17.01 ±0.11	12.45 ± 0.08	4.55 ± 0.04	2.7
rBAP 1	17.65 ± 0.24	13.74 ± 0.17	3.91 ± 0.08	3.5
rBAP 2	14.74 ± 0.45	11.14 ± 0.36	3.61 ± 0.10	3.1
BAP 0.5	9.63 ± 0.38	7.69 ± 0.07	2.04 ± 0.09	3.7
BAP 1	13.62 ± 0.78	10.56 ± 0.62	3.05 ± 0.16	3.5
BAP 2	8.50 ± 0.06	6.16 ± 0.05	2.33 ± 0.02	2.6

C—control, shoots cultivated on MS medium only with auxin (0.1 mg/L IAA), TPC—total polyphenol content, TPA—total polyphenolic acid content, TP—total phenylethanoid content.
